# Incidence and clinical predictors of cognitive decline in anticoagulated patients with atrial fibrillation: the Strat-AF Study

**DOI:** 10.1093/europace/euag168

**Published:** 2026-07-03

**Authors:** Antonio Di Carlo, Costanza Parenti, Eleonora Barucci, Giulia Salti, Emilia Salvadori, Giacomo Redi, Benedetta Formelli, Filippo Fratini, Anna Maria Gori, Giovanni Pracucci, Marzia Baldereschi, Francesco Meucci, Martina Berteotti, Francesco Alfano, Andrea Ginestroni, Enrico Fainardi, Rossella Marcucci, Anna Poggesi

**Affiliations:** Institute of Neuroscience, Italian National Research Council, Via Madonna del Piano, 10–50019 Florence, Italy; Institute of Neuroscience, Italian National Research Council, Via Madonna del Piano, 10–50019 Florence, Italy; NEUROFARBA Department, Neuroscience Section, University of Florence, Florence, Italy; Stroke Unit, Careggi University Hospital, Florence, Italy; Department of Biomedical and Clinical Sciences, University of Milan, Milan, Italy; Institute of Neuroscience, Italian National Research Council, Via Madonna del Piano, 10–50019 Florence, Italy; NEUROFARBA Department, Neuroscience Section, University of Florence, Florence, Italy; Stroke Unit, Careggi University Hospital, Florence, Italy; NEUROFARBA Department, Neuroscience Section, University of Florence, Florence, Italy; Department of Experimental and Clinical Medicine, University of Florence, Florence, Italy; Center for Atherothrombotic Diseases, Careggi University Hospital, Florence, Italy; NEUROFARBA Department, Neuroscience Section, University of Florence, Florence, Italy; Institute of Neuroscience, Italian National Research Council, Via Madonna del Piano, 10–50019 Florence, Italy; Structural Interventional Cardiology, Department of Clinical and Experimental Medicine, Careggi University Hospital, Florence, Italy; Department of Experimental and Clinical Medicine, University of Florence, Florence, Italy; Center for Atherothrombotic Diseases, Careggi University Hospital, Florence, Italy; Department of Experimental and Clinical Medicine, University of Florence, Florence, Italy; Center for Atherothrombotic Diseases, Careggi University Hospital, Florence, Italy; Neuroradiology Unit, Careggi University Hospital, Department of Experimental and Clinical Biomedical Sciences, University of Florence, Florence, Italy; Neuroradiology Unit, Careggi University Hospital, Department of Experimental and Clinical Biomedical Sciences, University of Florence, Florence, Italy; Department of Experimental and Clinical Medicine, University of Florence, Florence, Italy; Center for Atherothrombotic Diseases, Careggi University Hospital, Florence, Italy; NEUROFARBA Department, Neuroscience Section, University of Florence, Florence, Italy; Stroke Unit, Careggi University Hospital, Florence, Italy

**Keywords:** Atrial fibrillation, Cognitive impairment, Incidence, Vascular comorbidity

## Introduction

Atrial fibrillation (AF) affects ∼59 million individuals worldwide and is a recognized risk factor for cognitive decline.^1^ Possible mechanisms include embolism, silent brain infarctions, hypoperfusion, and inflammation.^2^ Despite the growing burden, longitudinal studies providing specific incidence rates are lacking.

The Stratification of Cerebral Bleeding Risk in AF (Strat-AF) Study investigated incidence and clinical predictors of cognitive impairment in older AF patients undergoing oral anticoagulants (OAC) using a comprehensive neuropsychological assessment.

## Methods

### Study design

Strat-AF is a prospective observational single-center study at Careggi University Hospital, Florence, Italy. Since September 2017, Strat-AF enrolled consecutive AF patients ≥65 years receiving OAC for thromboembolism prevention, without contraindications to magnetic resonance imaging (MRI), from the Atherothrombotic Diseases Outpatient Clinic.

Participants underwent clinical, functional, and neuropsychological assessments, plus brain MRI, at baseline and 18 months.^3^

Strat-AF2 (launched March 2021) extended follow-up to 36 months for the original cohort, adding new patients previously excluded for MRI refusal or contraindications. They underwent brain computed tomography, transthoracic echocardiography, cardiac MRI, and 18-month follow-up.

### Neuropsychological examination

Montreal Cognitive Assessment (MoCA) assessed baseline global cognition but was not used to define cognitive impairment. Six second-level tests evaluated memory (Rey Auditory-Verbal Learning/Short Story), attention/executive functions (Visual Search/Color Word Stroop), and language (Semantic Verbal Fluency/Sentence Construction). A score below the outer confidence limit for the 5^th^ percentile of Italian normative data (Equivalent Score = 0) on at least one second-level test defined cognitive decline.^3^

The study follows Helsinki Declaration and was approved by Careggi Ethics Committee. Participants provided written informed consent.

### Statistical analysis

Chi-square and *t*-tests compared categorical and continuous variables. Incidence rates of cognitive impairment were calculated per 1000 person-years. Person-years accrued from baseline to last follow-up for participants remaining normal and from baseline to the last normal visit plus half the interval to the first impaired visit for incident cases. Poisson distribution yielded 95% confidence intervals (CIs).

Logistic regression assessed non-participation. Independent predictors of cognitive impairment were identified using Cox proportional hazards models (hazards ratios with 95% CI) forcing age, sex, and education, and including variables with univariate *P* < 0.10. Kaplan–Meier curves and log-rank tests compared time-to-onset across vascular conditions and education.

Age and education were modelled continuously (1-year increment) but dichotomized for incidence and Kaplan–Meier analyses [age at median 74.8 years; education at 11 years (Italian compulsory schooling threshold)]. Tests are two-sided (*P* < 0.05).

## Results

Of 282 participants in Strat-AF (*n* = 194) and Strat-AF2 (*n* = 88), 137 had prevalent cognitive impairment and 5 lacked baseline evaluation, yielding 140 cognitively normal patients (mean age 76.8 ± 6.6 years, 60.7% male) at risk for incident impairment. Follow-up was achieved in 113 (80.7%), with 3 deaths (2.1%) and 24 withdrawals (17.2%). Attrition was associated with older age (odds ratio, 1.12; 95% CI, 1.04–1.20; *P* = 0.002) but not with sex or education.

Over a mean follow-up of 1.9 ± 1.1 years, totalling 214.5 person-years, 30 patients (26.5%) developed cognitive impairment, yielding an incidence rate of 139.9 cases per 1000 person-years (95% CI, 94.4–199.6).

**Figure 1:**
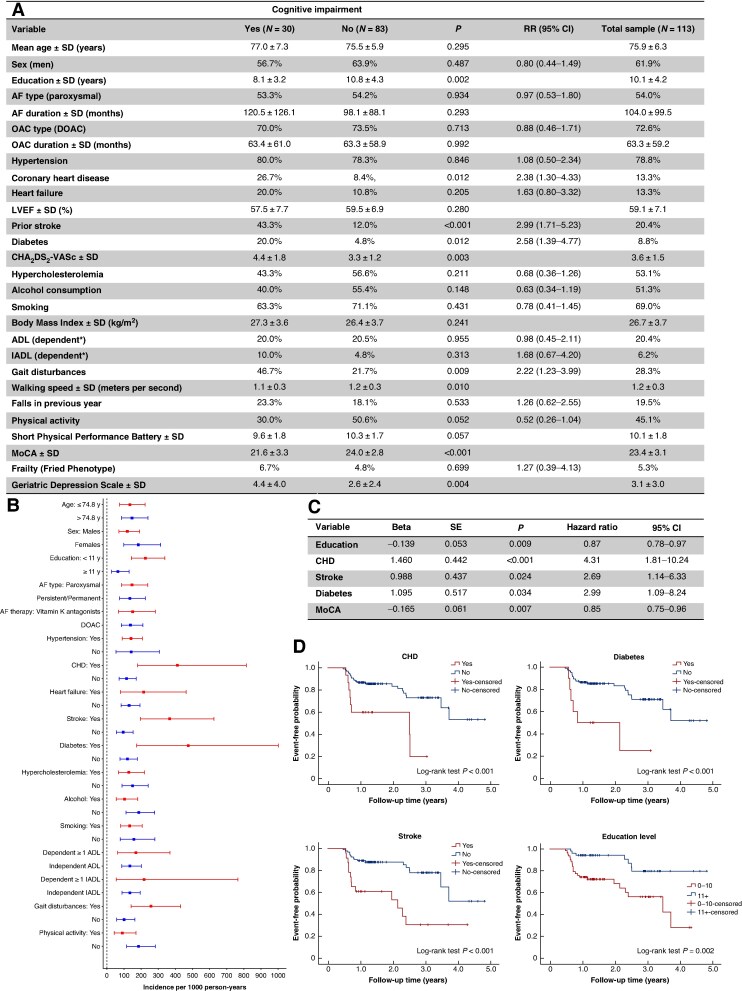
(*A*) Distribution of baseline variables by presence of cognitive impairment at follow-up and univariate relative risk for incident cases. (*B*) Incidence rates (per 1000 person-years) of cognitive impairment stratified by demographic characteristics, major vascular risk factors or diseases, and functional status. Education: lower (primary/lower secondary school) vs. higher (upper secondary/university) level. Vertical lines represent 95% Poisson confidence intervals. (*C*) Baseline variables significantly affecting the likelihood of developing cognitive impairment. Cox Proportional Hazards Multivariate Models including demographic characteristics, comorbidities, baseline global cognition, and function. (*D*) Kaplan–Meier plots of the probability of cognitive impairment during follow-up in patients with (red lines) and without (blue lines) a history of coronary heart disease, diabetes, or stroke and by education level. Abbreviations: ADL, activities of daily living; AF, atrial fibrillation; CHD, coronary heart disease; CI, confidence interval; DOAC, direct oral anticoagulants; IADL, Instrumental Activities of Daily Living (*dependent in at least one activity); LVEF, left ventricular ejection fraction; MoCA, Montreal Cognitive Assessment; OAC, oral anticoagulants; RR, relative risk; SD, standard deviation; SE, standard error.

*Figure 1A* illustrates baseline characteristics and relative risks for cognitive decline. Higher risk occurred with coronary heart disease (CHD), prior stroke, diabetes, and gait disturbances. Incident cases had significantly lower education and MoCA scores, slower gait, and higher CHA_2_DS_2_-VASc and Geriatric Depression Scale scores. The small difference in walking speed likely reflects minor variations in performance.

Incidence rates per 1000 person-years (*Figure 1B*) were substantially higher with lower education: 224.8 (95% CI, 142.5–337.3) vs. 62.4 (95% CI, 25.1–128.5); CHD: 412.4 (95% CI, 178.0–812.5) vs. 112.8 (95% CI, 70.7–170.7); and previous stroke: 364.9 (95% CI, 194.3–623.9) vs. 95.0 (95% CI, 55.4–152.2).

Multivariable Cox models (*Figure 1C*) identified CHD, prior stroke, and diabetes as independent predictors of cognitive impairment; higher education and baseline MoCA were protective. Kaplan–Meier curves and log-rank tests (*Figure 1D*) confirmed significantly greater risk with these conditions and low education.

## Discussion

We found high incidence of cognitive impairment in anticoagulated older AF patients, approximately twice the rate observed in general elderly populations.^4,5^ Independent predictors underscore the role of multimorbidity. Its impact on cognition is not simply additive, reflecting the specific combination and timing of conditions accumulated across the lifespan.^6^ This perspective aligns with evidence attributing about 40% of dementia cases to 12 potentially modifiable risk factors.^7^

Within this framework, the BRAIN-AF trial showed no benefit of low-dose rivaroxaban on cognitive decline, stroke, or transient ischemic attack in low-risk AF patients, indicating that the AF-cognition relationship extends beyond thromboembolism.^8^ Atrial fibrillation duration and burden, chronic hypoperfusion, inflammation, comorbidities, and the interplay with aging and genetic factors are likely key contributors.^9^

Small sample, single-centre design and tertiary outpatient setting may limit generalizability, particularly to AF populations with lower education, greater frailty, or reduced access to specialized care. Patients lost to follow-up were older, introducing potential bias. The modest number of incident events and the inclusion of several covariates resulted in a relatively low events-per-variable ratio, raising the possibility of model overfitting and reducing the precision of estimates. Sensitivity analyses using more parsimonious models yielded consistent results, but larger multicentre studies are needed. Anticoagulation adherence, dosing, and time in therapeutic range (vitamin K antagonists) were not collected. Suboptimal anticoagulation may have contributed to cognitive outcomes.

Although Strat-AF2 included patients without MRI to reduce neuroimaging-related selection bias, some selection toward a more stable outpatient population likely remains. Neuroimaging data were not analysed, representing another limitation. A dedicated analysis is ongoing.

Strengths include the first prospective estimation of cognitive decline incidence in older AF patients and the use of a comprehensive neuropsychological battery.

Cognitive decline was defined as impairment on at least one second-level test, both at baseline (excluding 137 patients) and at follow-up. This approach maximizes sensitivity for early changes and is consistent with DSM-5 criteria. However, it may reduce specificity and capture transient or clinically marginal abnormalities.^10^ Our incidence should therefore be interpreted with caution.

## Conclusions

Older AF patients on OAC showed high incidence of cognitive decline, significantly associated with vascular comorbidities but mitigated by education and baseline cognition. These findings support routine cognitive screening and comprehensive risk assessment. Early detection may enable tailored interventions.

## Data Availability

The data that support the findings of this study are available on reasonable request to the corresponding author. The data are not publicly available due to privacy or ethical restrictions.
